# Targeting the TGF-β Signaling Axis in Metastatic Colorectal Cancer: Where Do We Stand?

**DOI:** 10.3390/ijms242317101

**Published:** 2023-12-04

**Authors:** Kostas A. Papavassiliou, Donatella Delle Cave, Athanasios G. Papavassiliou

**Affiliations:** 1 First University Department of Respiratory Medicine, ‘Sotiria’ Hospital, Medical School, National and Kapodistrian University of Athens, 11527 Athens, Greece; konpapav@med.uoa.gr; 2 Institute of Genetics and Biophysics ‘Adriano Buzzati-Traverso’, CNR, 80131 Naples, Italy; 3 Department of Biological Chemistry, Medical School, National and Kapodistrian University of Athens, 11527 Athens, Greece

Colorectal cancer (CRC) represents the third most commonly diagnosed cancer and the second leading cause of cancer-related deaths worldwide [1]. It develops from the accumulation of multiple genetic and epigenetic alterations in the normal colonic and rectal epithelium, leading to the progression from colorectal adenomas to invasive carcinomas. Almost half of CRC patients will develop metastases in the course of the disease, and most patients with metastatic CRC are incurable. Particularly, the 5-year survival rate of patients with stage IV CRC at diagnosis is less than 10% [2]. In the metastatic setting, systemic treatment historically has consisted of chemotherapy (5-fluorouracil (5-FU), oxaliplatin, irinotecan, leucovorin, and capecitabine) in combination with biological agents (bevacizumab, cetuximab, and panitumumab). Nevertheless, major challenges, such as limited response, drug resistance, and systemic toxicity, lead to poor outcomes and survival rates for patients with metastatic CRC.

The emergence and increasing availability of molecular profiling are now pushing the field of CRC research toward a personalized biomarker-driven approach. Data emanating from the Cancer Genome Atlas project and other genomic studies have revealed that the pathobiology of CRC is based on a complex network of genetic and epigenetic alterations that result in the deregulation of numerous signaling pathways [3,4]. In turn, aberrant signaling contributes to tumor growth and metastatic dissemination and is providing new opportunities for drug development and treatment in CRC. Gene expression analysis, together with clinical features, has led to a novel classification of metastatic CRC into four molecular subtypes. One such subtype has been termed consensus molecular subtype 4 (CMS4), represents 23% of cases, and is characterized by a marked mesenchymal phenotype of CRC cells with prominent transforming growth factor-beta (TGF-β) signaling upregulation, stromal invasion, angiogenesis, unresponsiveness to treatment, and poor prognosis [4,5,6,7].

Accumulating evidence suggests that TGF-β signaling plays a central role in the progression of CRC, promoting epithelial-to-mesenchymal transition (EMT), angiogenesis, immunosuppression, stemness, and other malignant features [8,9]. Preclinical data from studies using models of metastatic CRC demonstrate that targeting of TGF-β signaling impedes metastasis [10,11,12,13]. Several strategies that modulate the TGF-β signaling cascade have been developed and are currently being evaluated in the preclinical and clinical setting. Therapeutic strategies against TGF-β signaling in CRC include: antisense oligonucleotides that inhibit the expression of TGF-β or its serine/threonine kinase receptor (TGF-βR) by binding to their mRNA and degrading it; integrin-blocking antibodies which function to block the integrin-mediated activation of the latent, inactive form of TGF-β; antibodies that hinder the physical interaction between TGF-β and TGF-βR either by binding and neutralizing TGF-β, or by binding and blocking TGF-βR type II (TGF-βRII); ligand traps which are engineered soluble forms of TGF-βRII that bind TGF-β and do not allow the latter to tether to the membrane-bound receptor; small-molecule inhibitors which selectively bind to the ATP-binding domain of TGF-βR and imbede its activity, thereby blocking the signaling pathway downstream of TGF-βR; and vaccine-based approaches that aim to downregulate the immunosuppressive aspect of TGF-β signaling [13].

The strategy of targeting TGF-β signaling-induced immunosuppression along with inhibitory immune checkpoints, such as cytotoxic T-lymphocyte-associated protein 4 (CTLA-4), programmed cell death protein 1/programmed death-ligand 1 (PD-1/PD-L1), lymphocyte-activation gene 3 (LAG-3), or T-cell immunoglobulin and mucin domain-containing molecule 3 (TIM-3), seems to be promising in metastatic CRC. In support of this concept, Tauriello et al. showed that blockade of TGF-β signaling in a mouse model of metastatic CRC by employing the small-molecule receptor kinase inhibitor galunisertib rendered tumors susceptible to PD-1/PD-L1 inhibitors [14]. Clinical trials (NCT03724851) are currently evaluating the therapeutic potential of vactosertib, a novel TGF-β1R type I (TGF-β1RI) kinase inhibitor, in combination with pembrolizumab in metastatic CRC, and their findings are eagerly awaited. Other promising therapeutic approaches related to TGF-β signaling in CRC include targeting small mothers against decapentaplegic (SMAD) proteins, which are transcription factors located downstream of the TGF-β signaling axis. Additionally, a novel potential therapeutic strategy in metastatic CRC is the targeting of TGF-β signaling via the drug pirfenidone. In a recent study, pirfenidone suppressed CRC cell proliferation and migration in vitro, as well as inhibited tumor growth, fibrosis, and inflammation in vivo in xenograft CRC models [15].

Preclinical and translational research indicates that blocking TGF-β signaling is a potentially effective therapeutic strategy in metastatic CRC, yet clinical application has been slow and, to date, not highly successful, as reflected by the fact that none of the TGF-β signaling inhibitors evaluated in clinical trials are currently approved for the management of advanced CRC. Since blockade of the TGF-β signaling pathway via single agents does not appear to result in direct CRC cell cytotoxicity, researchers and clinicians should consider combination strategies. In the era of cancer immunotherapy, combining TGF-β signaling blockade with immune checkpoint inhibitors represents an appealing therapeutic approach. Nevertheless, even with this drug combination, only a subset of metastatic CRC patients will respond, emphasizing the necessity of identifying clinically relevant predictive biomarkers by thoroughly analyzing the molecular features of metastatic CRC patients treated with anti-TGF-β signaling therapies. The TGF-β pathway deserves further translational investigation in the context of well-designed clinical trials where treatment selection is informed by multi-omic molecular profiling. Toward this goal, large-scale multi-omics data, analyzed by machine learning methods, could pave the way to successfully integrating the modulation of TGF-β signaling in the management of metastatic CRC.

## Data Availability

Data are contained within the article.
